# Effectiveness of the Cell-Based Quadrivalent Influenza Vaccine (SKYCellflu^®^ QIV) in Children and Adolescents: A Multicenter Test-Negative Case–Control Study in Korea

**DOI:** 10.3390/vaccines14010070

**Published:** 2026-01-08

**Authors:** Yoonsun Yoon, Hye Su Jeong, Kyeongmin Oh, Young June Choe, Hyun Mi Kang, Ji Young Park, Hye Young Kim, Yun-Kyung Kim

**Affiliations:** 1 Department of Pediatrics, Korea University Guro Hospital, Korea University College of Medicine, Seoul 08308, Republic of Korea; yhappy815@gmail.com; 2 Medical Affairs, SK bioscience Co., Ltd., Seongnam 13494, Republic of Korea; hyesujeong@sk.com (H.S.J.); ohkyeongmin@sk.com (K.O.); 3 Department of Pediatrics, Korea University Anam Hospital, Seoul 02841, Republic of Korea; choey@korea.ac.kr; 4 Department of Pediatrics, College of Medicine, The Catholic University of Korea, Seoul St. Mary’s Hospital, Seoul 06591, Republic of Korea; pedhmk@gmail.com; 5 Integrated & Respite Care Center for Children, Seoul National University Hospital, Seoul 03080, Republic of Korea; ji8303@gmail.com; 6 Department of Pediatrics, Korea University Ansan Hospital, Ansan 15355, Republic of Korea

**Keywords:** influenza, vaccine effectiveness, cell-based vaccine, pediatric, adolescent, case–control study

## Abstract

**Background**: Children and adolescents are pivotal in the transmission of influenza, and vaccination remains the most effective preventive measure. Cell-based influenza vaccines offer advantages over traditional egg-based vaccines by reducing egg-adapted mutations and improving antigenic match. SKYCellflu^®^ quadrivalent influenza vaccine (QIV; SK bioscience, Korea), the first cell-based QIV licensed in Korea for individuals aged 6 months and older, offers potential advantages; however, its real-world effectiveness in the Korean pediatric population remains limited. **Objective**: This study aimed to estimate the real-world effectiveness of SKYCellflu^®^ QIV, a cell-based QIV, in preventing laboratory-confirmed influenza among children and adolescents aged 6 months to 18 years in Korea during the 2024–2025 influenza season. **Methods**: A multicenter, prospective, test-negative case–control study was conducted from October 2024 to May 2025 across 25 institutions in Korea. Children and adolescents aged 6 months to 18 years who presented within 7 days of the onset of influenza-like illness (fever ≥ 38 °C and at least one respiratory symptom) were enrolled. Influenza infection was confirmed using rapid antigen tests or polymerase chain reaction; participants who tested positive were classified as cases, and those who tested negative for influenza served as controls. All participants were further categorized as vaccinated or unvaccinated based on receipt of SKYCellflu^®^ QIV. Those who received other influenza vaccines during the season were excluded. Vaccination status was verified through medical records and the national immunization registry. **Results**: A total of 1476 participants were included (751 cases, 725 controls). The overall adjusted vaccine effectiveness (aVE) was 45.57% (95% CI, 29.38–58.04). The vaccine demonstrated the highest effectiveness in children aged 6–35 months (aVE: 88.55%; 95% CI, 60.39–96.11). Effectiveness was higher against influenza B (aVE: 61.28%; 95% CI, 35.76–76.30) than influenza A (aVE: 41.63%; 95% CI, 22.55–56.01). The vaccine’s effectiveness in adolescents was not statistically significant due to the small sample size in this age group. **Conclusions**: This multicenter test-negative study provides the first real-world effectiveness of SKYCellflu^®^ QIV in a Korean pediatric population. The results suggest substantial protection in younger children, particularly against influenza B, and support the continued use of annual influenza vaccination in this population. Further studies with larger adolescent cohorts are needed to confirm these findings in older age groups.

## 1. Background

Influenza remains a major global public health burden, causing approximately 1 billion infections, 3–5 million cases of severe illness, and up to 650,000 respiratory deaths annually [1]. Vaccination is the most effective strategy for preventing influenza and its complications. Children experience higher rates of hospitalization and respiratory or neurologic sequelae than adults and may rarely develop severe manifestations such as influenza-associated acute myositis or myocarditis [2,3]. Pediatric vaccination additionally provides indirect community protection by reducing viral transmission [4].

In Korea, the National Immunization Program (NIP) has provided free influenza vaccination for children aged 6 months to 12 years since 2018, with expansion to 13 years of age in 2020. According to recent data from Korea’s NIP, pediatric influenza vaccination coverage has remained consistently around 70% over the past three years [5]. Recent real-world data from Korea show that pediatric influenza vaccine effectiveness (VE) has remained in the moderate range (approximately 30–50%). During the 2018–2019 season, a test-negative study reported a VE of 36.4% (95% CI, 13.9–53.1) in children, including 35.6% (95% CI, 10.5–53.7) in those aged 6 months to 12 years and 47.8% (95% CI, 25.9–63.3) among healthy children [6]. An earlier 2017–2018 analysis similarly estimated pediatric VE at approximately 53% [7].

Post-licensure evaluation of influenza VE is essential because clinical trial findings may not fully reflect performance under real-world conditions, and VE may vary across seasons due to antigenic drift. However, evidence in pediatric populations remains limited, as most VE studies have focused on adults or general populations.

As of December 2024, all influenza vaccines available in Korea are inactivated formulations, comprising 11 egg-based split vaccines and a cell culture-based quadrivalent subunit vaccine [8], cell-based vaccines, SKYCellflu^®^, may offer advantages over egg-based products, including avoidance of egg-adaptive mutations and potentially improved antigenic match [9,10].

This study aimed to assess the real-world effectiveness of the cell-based quadrivalent influenza vaccine (QIV) in preventing laboratory-confirmed influenza among children and adolescents aged 6 months to 18 years [11,12].

## 2. Methods

### 2.1. Study Design

This study employed a test-negative case–control (TNCC) design, recommended by the World Health Organization as a methodological standard for estimating influenza vaccine effectiveness in observational studies [13]. TNCC is widely used for influenza and other respiratory infections because it reduces bias related to healthcare-seeking behavior and illness severity and allows efficient use of existing testing infrastructure [14]. In this design, patients presenting with influenza-like illness (ILI) are tested for the influenza virus, and vaccination status is compared between those who test positive (cases) and those who test negative (controls).

### 2.2. Setting, Population

This multicenter study was conducted across 25 sites in Korea, including 5 tertiary hospitals and 20 clinics, from October 2024 through May 2025, corresponding to the 2024–2025 influenza season. Eligible participants were consecutively enrolled children and adolescents aged 6 months to 18 years who presented to participating facilities within 7 days of symptom onset during the national influenza epidemic alert period with ILI and had received at least one dose of SKYCellflu^®^ QIV(SK bioscience, Seongnam, Republic of Korea). Information on the number of vaccine doses and vaccination intervals was not collected. Participants were excluded if they lacked laboratory confirmation of influenza. Full eligibility criteria are presented in Supplementary Table S1.

Participants with influenza confirmed by rapid antigen test (RAT) or polymerase chain reaction (PCR) were classified as cases; those testing negative were classified as controls. Individuals who had received SKYCellflu^®^ QIV were categorized as vaccinated, whereas those who had not received any influenza vaccine were categorized as unvaccinated.

Vaccination history was verified through electronic medical records, official immunization records, and the National Immunization Registry. Receipt of SKYCellflu^®^ QIV was considered valid when administered on or after 1 July 2024, and when ILI onset occurred more than 14 days after vaccination. Participants with ILI onset within 14 days of vaccination were classified as unvaccinated. Re-enrollment was permitted if a new ILI episode occurred more than 30 days after the initial diagnosis and was confirmed to be caused by a different influenza strain.

Baseline characteristics included age, sex, geographic region, timing of ILI onset and specimen collection, type of healthcare facility, and interval since vaccination. ILI was defined according to the Korea Disease Control and Prevention Agency (KDCA) criteria as fever ≥ 38 °C plus at least one respiratory symptom (cough, sore throat, rhinorrhea, or nasal congestion) [15].

### 2.3. Sample Size Calculation

The required sample size was estimated based on the influenza vaccination rate (88.2% among controls) and VE (51.1%) among Korean children and adolescents aged 6 months to 18 years, as reported by the KDCA [16]. Using the absolute precision formula [17] with an assumed precision of 0.3 and a case-to-control ratio of 1:3, a total of 1564 participants (391 cases and 1173 controls) were required.

### 2.4. Outcome

The primary outcome was the VE of SKYCellflu^®^ QIV against all influenza types during the 2024–2025 season. Secondary outcomes included (1) VE stratified by age group—6 months to 13 years (the age group covered by the Korean NIP) [12], which was further subdivided into 6–35 months and 36 months to 13 years—and 14 to 18 years; and (2) VE by influenza type (A and B).

### 2.5. Statistical Analysis

Continuous variables were summarized using descriptive statistics, and categorical variables were reported as frequencies and percentages. Between-group comparisons of continuous variables were conducted using the t test when normality was satisfied; otherwise, the Wilcoxon rank sum test was applied. For categorical variables, the chi-squared test was used, and the Fisher exact test was applied when more than 20% of cells had an expected frequency < 5.

Crude VE was calculated as (1 − odds ratio [OR]) × 100 with corresponding 95% confidence intervals (CIs). Adjusted VE (aVE) was estimated using logistic regression, including age group, sex, geographic region, presence of underlying medical conditions, and timing of specimen collection as covariates. All tests were two-sided with a significance threshold of *p*-value < 0.05. Analyses were performed using SAS software version 9.4 (Enterprise BI Server; SAS Institute Inc., Cary, NC, USA).

### 2.6. Ethical Statement

Each of the 5 tertiary hospitals obtained approval from their respective institutional review boards (IRB), and the 20 participating clinics without independent IRB were covered under the approval of an IRB-affiliated institution. Written informed consent was obtained from legal guardians, and assent was obtained from children and adolescents when appropriate, in accordance with the Declaration of Helsinki and national regulations.

## 3. Results

### 3.1. Baseline Characteristics of the Study Participants

A total of 1566 participants with ILI were screened for eligibility. After excluding 90 individuals who were ineligible (n = 77), withdrew consent (n = 1), or met other exclusion criteria (n = 12), 1476 participants were included in the final analytic cohort. Among these, 751 had laboratory-confirmed influenza by RAT or PCR and were classified as cases, while 725 tested negative and were classified as controls (Supplementary Figure S1).

Participant characteristics are summarized in Table 1 and Supplementary Table S2. Most participants were aged 6 months to 13 years (cases, 77.90%; controls, 82.34%), and the sex distribution was comparable between groups (male: cases, 50.07%; controls, 49.93%). Among cases, ILI onset occurred predominantly in December (52.86%) and January (36.22%), and specimen collection followed a similar temporal pattern (December, 51.40%; January, 37.55%). Controls showed analogous seasonal distributions, with the highest frequencies observed in January (30.48%) and December (28.00%). Most participants were evaluated in outpatient settings (cases, 98.14%; controls, 97.79%). RAT was the primary diagnostic modality used (cases, 99.47%; controls, 98.90%). Among influenza-positive specimens, influenza A predominated (82.96%), whereas influenza B accounted for 17.31%. Among vaccinated cases, the majority (84.18%) had received vaccination 0.5 to less than 3 months before illness onset.

### 3.2. Vaccine Effectiveness During the 2024–2025 Season

Overall, 297 of 751 cases (39.55%) and 412 of 725 controls (56.83%) were vaccinated. Vaccination coverage was higher among children aged 6 months to 13 years (cases, 275 of 585 [47.01%]; controls, 399 of 597 [66.83%]) than among adolescents aged 14 to 18 years (cases, 22 of 166 [13.25%]; controls, 13 of 128 [10.16%]).

VE estimates for the study period are summarized in Table 2. The overall aVE was 45.57% (95% CI, 29.38–58.04). Among children aged 6 months to 13 years, aVE reached 57.64% (95% CI, 44.41–67.73). Infants and young children aged 6 to 35 months demonstrated the highest protection, with an aVE of 88.55% (95% CI, 66.30–96.11), whereas those aged 36 months to 13 years showed a lower aVE of 46.98% (95% CI, 28.90–60.47). In contrast, adolescents aged 14 to 18 years had an aVE of 61.66% (95% CI, −270.51 to 29.47), which was not statistically significant.

When stratified by influenza type, the aVE against influenza B was 61.28% (95% CI, 36.75–76.30), exceeding the estimate observed for influenza A (41.63%; 95% CI, 22.55–56.01). This trend was consistent among children aged 6 months to 13 years, with aVE values of 66.95% (95% CI, 45.35–80.01) for influenza B and 55.89% (95% CI, 40.89–67.08) for influenza A.

**Table 2:** Crude and adjusted vaccine effectiveness.

	Case, n (%) (N = 751)		Control, n (%) (N = 725)		Crude VE % (95% CI)	Adjusted VE % (95% CI)	*p*-Value
	No. Vaccination	Total	No. Vaccination	Total			
**Overall**	297 (39.55)	751	412 (56.83)	725	50.30 (38.85, 59.61)	45.57 (29.38, 58.04)	<0.0001
**Age group**							
6 months–13 years	275 (47.01)	585	399 (66.83)	597	55.98 (44.30, 65.21)	57.64 (44.41, 67.73)	<0.0001
6 months–35 months	13 (46.43)	28	116 (77.33)	150	74.60 (41.44, 88.98)	88.55 (66.30, 96.11)	<0.0001
36 months–13 years	262 (47.04)	557	283 (63.31)	447	48.53 (33.63, 60.09)	46.98 (28.90, 60.47)	<0.0001
14–18 years	22 (13.25)	166	13 (10.16)	128	−35.15 (−179.92, 34.75)	−61.66 (−270.51, 29.47)	0.2564
**Influenza A**							
Overall	261 (41.89)	623	412 (56.83)	725	45.23 (31.98, 55.89)	41.63 (22.55, 56.01)	0.0002
6 months–13 years	243 (48.89)	497	399 (66.83)	597	52.52 (39.35, 62.84)	55.89 (40.89, 67.08)	<0.0001
14–18 years	18 (14.29)	126	13 (10.16)	128	−47.44 (−215.32, 31.06)	−197.23 (−779.27, −0.48)	0.0490
**Influenza B**							
Overall	37 (28.46)	130	412 (56.83)	725	69.77 (54.53, 79.91)	61.28 (36.75, 76.30)	0.0002
6 months–13 years	33 (36.67)	90	399 (66.83)	597	71.27 (54.43, 81.89)	66.95 (45.35, 80.01)	<0.0001
14–18 years	4 (10.00)	40	13 (10.16)	128	1.70 (−220.37, 69.84)	45.62 (−128.92, 87.08)	0.4061

Abbreviations: CI, confidence interval; VE, vaccine effectiveness. Adjusted for age, sex, region, presence of underlying conditions, and month of specimen collection.

## 4. Discussion

This multicenter observational study evaluated the real-world effectiveness of the cell-based influenza vaccine (SKYCellflu^®^ QIV), during the 2024–2025 influenza season in Korea. Significant overall VE was observed, with particularly strong protection in children aged 6 months to 13 years, the age group covered by the Korean NIP [12].

In this study, the overall aVE of SKYCellflu^®^ QIV was 45.57% (95% CI, 29.38–58.04). Considering that most participants were outpatients, this estimate was comparable to those reported in children and adolescent populations during the same 2024–2025 influenza season. In the United States (US), Centers for Disease Control and Prevention data for individuals aged <18 years—including outpatients and encompassing all vaccine types—showed overall aVE estimates ranging from 32% (95% CI, 1–54) to 60% (95% CI, 56–63) [18]. Similarly, in the United Kingdom, an analysis of primary care patients aged 2–17 years, also including all vaccine types, reported an aVE of 55.6% (95% CI, 48.7–61.5) [19]. Comparable findings have been reported in studies evaluating the same inactivated QIV. A Japanese outpatient study conducted during the 2015–2016 season among 4409 children aged 6 months to <15 years reported an aVE of 49.0% (95% CI, 42.0–55.0) [20], whereas an Australian inpatient study conducted during the 2017 season among 1268 children aged <16 years found an aVE of 30.3% (95% CI, 2.6–50.2) [21]. Variations in the study season, population characteristics (outpatient vs. inpatient), and vaccine formulation (QIV vs. all vaccine types) may account for the observed differences across studies. Taken together, the aVE observed for SKYCellflu^®^ QIV in this Korean study appears broadly consistent with previously reported influenza VE across diverse seasons and clinical settings.

Additional evidence from Korea supports this interpretation. A Korean study conducted from 2014 to 2016 among children aged 6 months to <5 years reported an aVE of 38.4% for seasonal influenza vaccines [22]. Another Korean study during the 2018–2019 season among children aged 6 months to <12 years found an aVE of 36.4% (95% CI, 13.9–53.1) [6]. Although these studies evaluated all seasonal vaccine types rather than exclusively egg-based or cell-based formulations, their estimates fall within a similar range, further indicating that the VE observed in this study was consistent with previously reported influenza vaccine performance in Korean pediatric populations.

The markedly higher aVE observed in children aged 6 to 35 months (88.55%; 95% CI, 66.30–96.11) exceeded estimates from comparable studies. In Guangdong Province, China (2021–2024), aVE among children aged 6 to 35 months who received live-attenuated, trivalent, or quadrivalent inactivated vaccines was 32% (95% CI, 19–43), with a vaccine coverage of only 10.3% [23]. Similarly, in Andalusia, Spain (2022–2023), an aVE of 54.0% (95% CI, 13.0–77.6) was reported for inactivated QIV against influenza-related hospitalization among children aged 6–23 months, with a vaccination coverage of 45.3% [24]. In contrast, in Korea, children are routinely vaccinated under the NIP, with coverage reaching 80.6% among those aged 1 to 5 years [25]. Variations in VE across studies may reflect differences in vaccine formulation, population characteristics, and coverage levels.

Younger children generally exhibit higher influenza VE than older children or adults because they have limited prior exposure to influenza viruses and therefore minimal immune imprinting [26]. Their immune responses are shaped predominantly by current vaccine antigens rather than by preexisting strain-biased memory B cells that can attenuate VE in older age groups. Among children aged under 36 months, cell-based influenza vaccines may confer particularly high effectiveness because they avoid egg-adaptive mutations and maintain closer antigenic similarity to circulating viruses [27]. In this influenza-naïve age group, such improved antigenic fidelity likely supports robust de novo antibody responses, contributing to the higher VE observed. Beyond immunological factors, developmental and behavioral factors may also influence age-specific VE. Infants have immature immune systems with incomplete T-cell-independent B-cell activation [28], whereas older children and adolescents experience hormonal changes and behavioral patterns that may alter exposure risk and immune modulation [29]. These differences should be considered when interpreting VE across age groups.

In this study, the aVE against influenza B was 61.28% (95% CI, 36.75–76.30), which was significantly higher than that against influenza A (41.63%; 95% CI, 22.55–56.01). During the 2024–2025 influenza season in Korea, two epidemic peaks were observed: an initial wave dominated by influenza A in January, followed by a second peak with increased influenza B detections in March [30]. Consistent with this pattern, most specimens in our study were collected during the first peak, and most influenza-positive cases were type A (82.96%); however, the aVE was nevertheless higher against type B. This finding may reflect the intrinsic advantages of cell-based QIV, which avoids egg-adaptive mutations and thus maintains higher antigenic fidelity with circulating B strains [10], as similarly demonstrated in a US study reporting superior VE of cell-based vaccine against influenza B compared with egg-based vaccines [31]. Lower VE for influenza A during A (H3N2)-dominant seasons has also been attributed to host–virus interactions and prior antigenic exposures [32]. Korean VE estimates from the same 2024–2025 season also showed markedly reduced effectiveness against influenza A (0.4%; 95% CI, −33.2 to 25.5), supporting the possibility that strain mismatch contributed to the lower A-type VE observed in this study [33].

In this study, the aVE among adolescents aged 14 to 18 years was not statistically significant. This finding is likely attributable to the low vaccination coverage in this age group. In Korea, influenza vaccination is included in the NIP and provided free of charge to children aged 6 months to 13 years, with a reported coverage of 69.5% [5]. However, coverage declines sharply during adolescence, reaching only 33.0% among those aged 15 to 18 years [25]. A similarly suboptimal coverage pattern has been reported internationally, with rates of 50.8% among US adolescents aged 13 to 17 years [34] and 35.1% among those aged 12 to 17 years in Hong Kong [35], indicating that insufficient vaccine uptake in this population is a widespread challenge. In the present study, vaccination coverage was even lower (13.25% [22/166] among cases and 10.16% [13/128] among controls). Shinjoh et al. (2025) [36] likewise reported difficulty in estimating VE due to limited vaccination uptake, noting that such low coverage levels may result in insufficient case numbers and imbalanced sample sizes between vaccinated and unvaccinated groups, thereby contributing to sparse data bias and limiting the ability to obtain statistically robust estimates. Notably, epidemiological surveillance during the 2024–2025 season in Korea identified school-aged children and adolescents as the main drivers of community transmission [30]; in this context, the limited number of vaccinated adolescents observed in our study underscores the importance of considering this age group in influenza vaccination policy discussions.

This study has several limitations. First, participants were recruited from selected regions, introducing potential selection bias. Second, although the sample size was planned assuming a 1:3 case–control ratio, most participants were enrolled during the first epidemic peak—when influenza positivity exceeded 70% [30]—resulting in an actual ratio near 1:1 and reduced statistical power. Small sample sizes or low vaccination coverage in key subgroups (particularly those aged 6–35 months and 14–18 years) further limited the precision of VE estimates, as reflected by wide confidence intervals. Third, the lower sensitivity of RAT compared to PCR may have introduced misclassification bias in the test-negative design, potentially leading to an underestimation of VE. Thus, the VE estimates should be interpreted as conservative. Despite these limitations, this study also has several strengths. To the best of our knowledge, this is the first real-world evidence study in Korea evaluating the effectiveness of a cell-based QIV among children and adolescents. By focusing on these age groups—often difficult to recruit in conventional clinical trials—this study provides VE estimates under a routine clinical setting. In addition, this single-season analysis reflects the influenza season following epidemiological shifts associated with the COVID-19 pandemic, offering insights into VE under contemporary circulation patterns.

## 5. Conclusion

This study represents the first real-world evidence evaluating SKYCellflu^®^ QIV, a cell-based QIV in children and adolescents in Korea, demonstrating clinically meaningful VE during the 2024–2025 influenza season. These findings support the integration into broader NIP strategies of cell-based influenza vaccines in children and adolescent populations and highlight their potential value in guiding national immunization strategies and public health policy.

## Figures and Tables

**Table 1:** Baseline characteristics of the study population.

	Case, n (%) (N = 751)	Control, n (%) (N = 725)	Total, n (%) (N = 1476)	*p*-Value
**Age Group**				0.0324
6 months–13 years	585 (77.90)	597 (82.34)	1182 (80.08)	
6 months–35 months	28 (3.73)	150 (20.69)	178 (12.06)	
36 months–13 years	557 (74.17)	447 (61.66)	1004 (68.02)	
14–18 years	166 (22.10)	128 (17.66)	294 (19.92)	
**Sex**				0.9585
Male	376 (50.07)	362 (49.93)	738 (50.00)	
Female	375 (49.93)	363 (50.07)	738 (50.00)	
**Region**				<0.0001
Seoul	148 (19.71)	268 (36.97)	416 (28.18)	
Gyeonggi-do	484 (64.45)	373 (51.45)	857 (58.06)	
Others	119 (15.85)	84 (11.59)	203 (13.75)	
**Type of visit**				0.6367 *
Outpatient	737 (98.14)	709 (97.79)	1446 (97.97)	
Emergency	3 (0.40)	6 (0.83)	9 (0.61)	
Inpatient	11 (1.46)	10 (1.38)	21 (1.42)	
**Test type**				<0.0001
RAT	747 (99.47)	717 (98.90)	1464 (99.19)	
PCR	11 (1.46)	8 (1.10)	19 (1.29)	
**Influenza Strain**				N/A
** Influenza A ^†^**	623 (82.96)	-	623 (42.21)	
Result of Influenza A ^‡^				
Negative	129	725	854	
Positive	629	-	629	
** Influenza B ^†^**	130 (17.31)	-	130 (8.81)	
Result of Influenza B ^‡^				
Negative	627	725	1352	
Positive	131	-	131	
**Time since vaccination**				N/A
0.5–<3 months	250 (84.18)	-	250 (35.26)	
3–<6 months	42 (14.14)	-	42 (5.92)	
≥6 months	5 (1.68)	-	5 (0.71)	

Abbreviations: RAT, rapid antigen test; PCR, polymerase chain reaction; N/A, not applicable. Others include Chungcheongnam-do, Busan. * Fisher exact test. ^†^ Defined as participants with a positive result from either RAT or PCR. Two participants were diagnosed with both influenza A and B. ^‡^ Indicates the number of RAT and PCR test results (i.e., test counts), not the number of participants. Note: Percentages were calculated using the number of participants in each group as the denominator; for “time since vaccination,” the denominator was the number of participants who received vaccination.

## Data Availability

The data are not publicly available due to privacy and ethical restrictions, but are available on reasonable request from the corresponding authors.

## Supplementary Materials

The following supporting information can be downloaded at: https://www.mdpi.com/article/10.3390/vaccines14010070/s1, Figure S1: Flowchart of Participant Screening and Classification; Table S1: Inclusion and Exclusion Criteria of study population; Table S2: Additional Baseline Characteristics of the Study Population.
