# Supplementary material for: Effectiveness of the Cell-Based Quadrivalent Influenza Vaccine (SKYCellflu® QIV) in Children and Adolescents: A Multicenter Test-Negative Case–Control Study in Korea
Source: Vaccines (Basel). 2026 Jan 8;14(1):70. doi: 10.3390/vaccines14010070 (PMC12846570; doi:10.3390/vaccines14010070)
Supplement: Supplementary file 1 [file vaccines-14-00070-s001.zip › vaccines-4088106-supplementary.pdf]

Supplementary Table S1. Inclusion and Exclusion Criteria of study population.

**Inclusion Criteria**	
1.	Children and adolescents aged 6 months to 18 years
2.	Presentation to a participating hospital or clinics, or documentation of a visit, within 7 days of symptom onset with ILI during the national influenza epidemic alert period
3.	Provision of written informed consent by a legal guardian and assent by the participant, when applicable, after full explanation and understanding of the study
**Exclusion Criteria**	
1.	No available influenza test results (RAT or PCR) within 7 days of ILI symptom onset
2.	Receipt of any influenza vaccine other than SKYCellflu® QIV after July 1 of the corresponding influenza season
3.	Inability to verify vaccination history for the current influenza season through vaccination records (electronic medical records, official immunization records, and the National Immunization Registry) or unclear documentation
4.	Use of, or planned use of, antiviral agents within 4 weeks prior to RAT or PCR testing (excluding topical antivirals)
5.	Any other medical reason deemed by the investigator to make the participant unsuitable for study participation

Abbreviations: ILI, influenza-like illness; RAT, rapid antigen test; PCR, polymerase chain reaction; QIV, quadrivalent influenza vaccine

Supplementary Figure S1. Flowchart of Participant Screening and Classification.

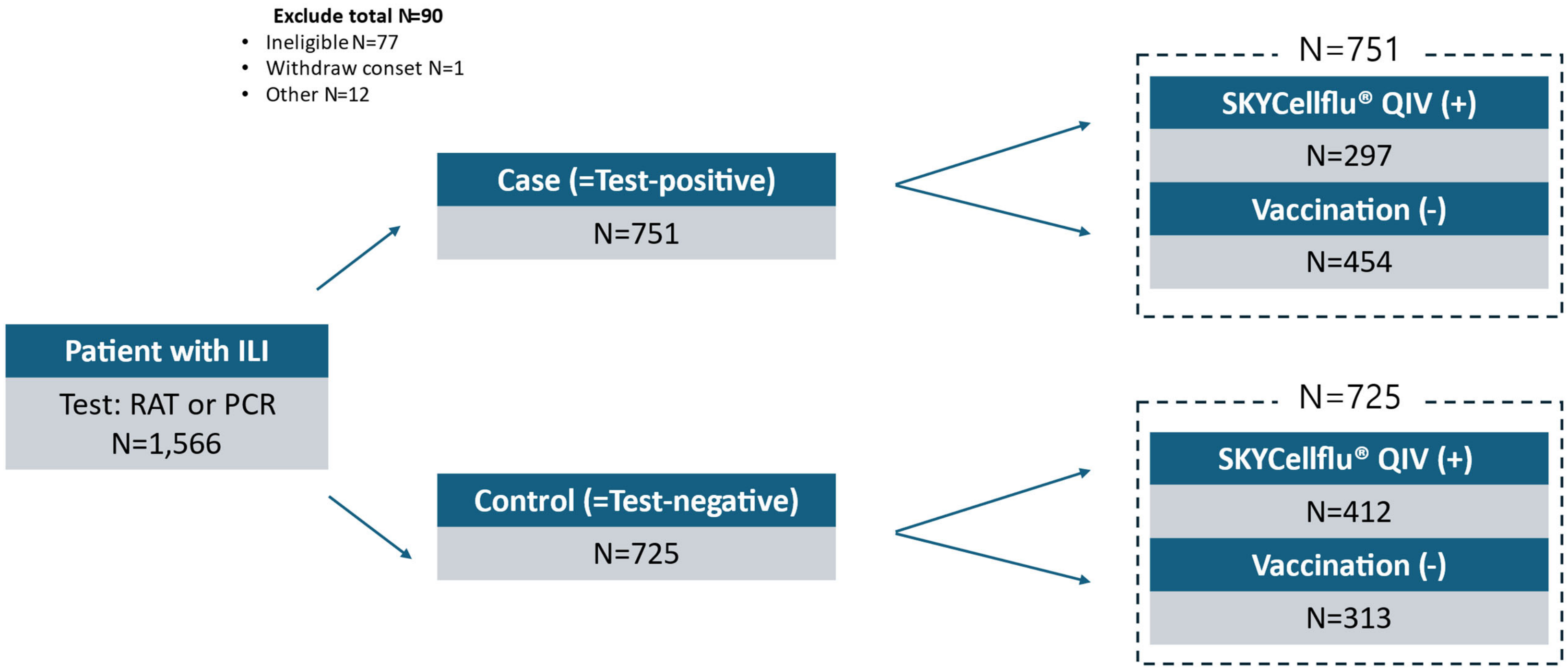

Abbreviations : ILI, influenza-like illness; RAT, rapid antigen test; PCR, polymerase chain reaction; QIV, Quadrivalent Influenza Vaccine

Supplementary Table S2. Additional Baseline Characteristics of the Study Population.

	Case, n (%) (N=751)	Control, n (%) (N=725)	Total, n (%) (N=1,476)	P-value
**Onset of ILI symptom**				<0.0001
October	0 (0.00)	6 (0.83)	6 (0.41)	
November	5 (0.67)	101 (13.93)	106 (7.18)	
December	397 (52.86)	203 (28.00)	600 (40.65)	
January	272 (36.22)	221 (30.48)	493 (33.40)	
February	20 (2.66)	75 (10.34)	95 (6.44)	
March	28 (3.73)	60 (8.28)	88 (5.96)	
April	28 (3.73)	52 (7.17)	80 (5.42)	
May	1 (0.13)	7 (0.97)	8 (0.54)	
**Underlying condition**				0.5049
Yes	489 (65.11)	484 (66.76)	973 (65.92)	
No	262 (34.89)	241 (33.24)	503 (34.08)	
**Specimen collection timing**				<0.0001
October	0 (0.00)	2 (0.28)	2 (0.14)	
November	5 (0.67)	104 (14.34)	109 (7.38)	
December	386 (51.40)	203 (28.00)	589 (39.91)	
January	282 (37.55)	220 (30.34)	502 (34.01)	
February	21 (2.80)	76 (10.48)	97 (6.57)	
March	28 (3.73)	61 (8.41)	89 (6.03)	
April	28 (3.73)	49 (6.76)	77 (5.22)	
May	1 (0.13)	10 (1.38)	11 (0.75)	

Abbreviations: ILI, influenza-like illness
